# *GBA1* as a risk gene for osteoporosis in the specific populations and its role in the development of Gaucher disease

**DOI:** 10.1186/s13023-024-03132-x

**Published:** 2024-04-04

**Authors:** Chung-Hsing Wang, Yu‐Nan Huang, Wen-Ling Liao, Ai-Ru Hsieh, Wei-De Lin, Kai-Wen Liu, Wen-Li Lu, Chieh‐Chen Huang, Yin-Hsiu Chien, Ni-Chung Lee, Pen-Hua Su, Fuu-Jen Tsai

**Affiliations:** 1 Division of Genetics and Metabolism, Children’s Hospital of China Medical University, Taichung, Taiwan; 2 School of Medicine, China Medical University, Taichung, Taiwan; 3 Department of Pediatrics, Chung Shan Medical University Hospital, No. 110, Sec. 1, Jianguo N. Rd., South Dist., Taichung, 402306 Taiwan; 4 School of Medicine, Chung Shan Medical University, Taichung, Taiwan; 5 Department of Life Sciences, National Chung-Hsing University, Taichung, Taiwan; 6 Graduate Institute of Integrated Medicine, College of Chinese Medicine, China Medical University, Taichung, Taiwan; 7 Department of Medical Research, Center for Personalized Medicine, China Medical University Hospital, Taichung, Taiwan; 8 Department of Statistics, Tamkang University, New Taipei City, Taiwan; 9 Department of Medical Research, Genetic Center, China Medical University Hospital, No. 2 Yuh-Der Road, Taichung, 404 Taiwan; 10 School of Post Baccalaureate Chinese Medicine, China Medical University, Taichung, Taiwan; 11 Department of Pediatrics, National Taiwan University Hospital, Taipei, Taiwan

**Keywords:** Gaucher disease, GBA1, Osteoclastogenesis, Inflammasome, Endoplasmic reticulum stress, Osteoporosis

## Abstract

**Background:**

Osteoporosis and its primary complication, fragility fractures, contribute to substantial global morbidity and mortality. Gaucher disease (GD) is caused by glucocerebrosidase (GBA1) deficiency, leading to skeletal complications. This study aimed to investigate the impact of the GBA1 gene on osteoporosis progression in GD patients and the specific populations.

**Methods:**

We selected 8115 patients with osteoporosis (T-score ≤ − 2.5) and 55,942 healthy individuals (T-score > − 1) from a clinical database (*N* = 95,223). Monocytes from GD patients were evaluated in relation to endoplasmic reticulum (ER) stress, inflammasome activation, and osteoclastogenesis. An in vitro model of GD patient’s cells treated with adeno-associated virus 9 (AAV9)-GBA1 to assess GBA1 enzyme activity, chitotriosidase activity, ER stress, and osteoclast differentiation. Longitudinal dual-energy X-ray absorptiometry (DXA) data tracking bone density in patients with Gaucher disease (GD) undergoing enzyme replacement therapy (ERT) over an extended period.

**Results:**

The GBA1 gene variant rs11264345 was significantly associated [*P* < 0.002, Odds Ratio (OR) = 1.06] with an increased risk of bone disease. Upregulation of Calnexin, NOD-, LRR- and pyrin domain-containing protein 3 (NLRP3) and Apoptosis-associated speck-like protein containing a C-terminal caspase recruitment domain (ASC) was positively associated with osteoclastogenesis in patients with GD. In vitro AAV9-GBA1 treatment of GD patient cells led to enhanced GBA1 enzyme activity, reduced chitotriosidase activity, diminished ER stress, and decreased osteoclast differentiation. Long-term bone density data suggests that initiating ERT earlier in GD leads to greater improvements in bone density.

**Conclusions:**

Elevated ER stress and inflammasome activation are indicative of osteoporosis development, suggesting the need for clinical monitoring of patients with GD. Furthermore, disease-associated variant in the GBA1 gene may constitute a risk factor predisposing specific populations to osteoporosis.

**Supplementary Information:**

The online version contains supplementary material available at 10.1186/s13023-024-03132-x.

## Background

The human skeleton acts as a dynamic organ that undergoes continuous remodeling throughout an individual’s life in response to various environmental stimuli [1]. Bone remodeling occurs through the coordinated activity of osteoclasts that break down old bone, and osteoblasts that form new bone. Osteoclasts are specialized monocyte/macrophage cells that adhere to the bone matrix and secrete degrading enzymes [2, 3].

Gaucher disease (GD; OMIM #230800), a rare hereditary lysosomal disorder involving lipid accumulation and the malfunction of various organs, is caused by disease-associated variants in *glucocerebrosidase* (*GBA1*; also known as GCase), leading to GBA1 protein deficiency in lysosomes [4]. GCase deficiency leads to glucosylceramide accumulation in the lysosomes of the monocyte/macrophage system in the bone marrow, liver, and spleen, resulting in cytopenia, hepatosplenomegaly, and significant bone symptoms [5]. Three GD subgroups have been identified based on the presence [Type 2 (GD2) or Type 3 (GD3)] or absence [Type 1 (GD1)] of neurological symptoms. Significant clinical complications in GD1/GD3 include bone disease, such as avascular necrosis (AVN), bone infarct (BI), or pathological fracture [6, 7].

The endoplasmic reticulum (ER), which widely distributed in the cytoplasm, regulates cellular homeostasis by forming connections with multiple other organelles [8]. Mutant proteins are detected as misfolded by the ER quality management machinery and maintained in the ER, from which they are retrotranslocated back to the cytosol to be destroyed by the ubiquitin–proteasome pathway after a specific amount of time has elapsed, during which the ER chaperones attempt to refold the proteins [9]. Misprocessing of mutant GBA1 in the ER during differentiation into dopaminergic neurons has been linked to ER stress activation and aberrant cellular lipid profiles [10]. Accumulating evidence suggests that ER stress can induce the activation of inflammasomes and contribute to the development of various diseases, including neurodegenerative and metabolic disorders [11, 12]. Although osteoclasts play a critical role in maintaining both health and bone disease, further studies are required to understand the ER–inflammasome interactions and the contribution of the *GBA1* disease-associated variant to the development of bone disease in patients with GD and the specific populations.

## Materials and methods

### Specific populations and GD patients

To analyze *GBA1* variants in the specific populations, we analyzed sequence data from the Taiwan Biobank (TWB) database, includes 95,223 individuals aged 30–70 years with T-score data. To elucidate the intracellular molecular mechanism underlying the association between *GBA1* variants and osteoporosis, we isolated monocytes from six GD patients and analyzed their ER stress indicators, inflammasome markers, and osteoclast differentiation levels. These patients had a documented history of osteoporosis.

### Database preparation and osteoporosis status definition

We defined osteoporosis based on hip bone mineral density (BMD) assessed using dual-energy X-ray absorptiometry (DXA). Participants were classified into the osteoporosis group if their T-score was ≤ − 2.5, and into the non-osteoporosis group if their T-score was > − 1. The TWB database, containing genetic data for 95,233 individuals, was utilized. Since 2013, TWB has enrolled individuals from the broader Taiwanese population, specifically those of Taiwanese Han-Chinese ethnicity, aged 30–70, without a history of cancer. TWB collects samples and pertinent data at enrollment and conducts follow-ups every 2–4 years. This database includes demographic, health habits, physical/laboratory examination results, and genetic data. The study received approval from the Institutional Review Board and Ethics Committee of CMUH (IRB no. CMUH109-REC1-003) and Taiwan Biobank (approval no. TWBR10905-04). Informed consent was obtained in accordance with the Declaration of Helsinki, and participants could withdraw consent at any time [13].

### Genetic analysis and quality control

Individuals from the TWB database were included in this study. Genotyping was performed using the special TWB chips (Affymatrix TWB v.1 and Affymatrix TWB v.2) and an Axiom Genome-Wide Array Plate System (Affymetrix, Santa Clara, California) [14]. Following the procedures outlined in earlier investigations, samples and single-nucleotide polymorphisms (SNPs) were used for genotyping and quality control checks [14]. Using GENCODE version 38, 17 SNPs were identified in the *GBA1* coding regions of the TWB chips (Additional file 1). Low call rate (information: 99%), minor allele frequency (< 0.05), Hardy–Weinberg equilibrium in controls (*P* < 0.0005), and SNPs with low imputation quality (information < 0.3) were excluded from the analysis.

### Phenome-wide association studies (PheWAS) analysis of *GBA1* rs1126434

PheWAS analysis utilized genotype (rs11264345) data and 41 health outcome metrics from TWB. We employed linear and logistic regression models to assess the SNP's association with each of the 31 quantitative and 10 binary traits, ensuring statistical power by including phenotypes with more than 200 cases. Phenotypic cases were defined by at least two diagnoses corresponding to the specific Phecode, while controls had no Phecode diagnosis or met Phecode exclusion criteria [15]. The characteristics of the PheWAS in this study are recorded in Additional file 2.

### Clinical participants

Blood samples were collected from six patients with GD and six healthy nonhospitalized volunteers [matched for age, sex, and body mass index (BMI)] from China Medical University Children's Hospital Medical Center, who had not shown any symptoms of concurrent infection in the preceding 6 months. All participants were recruited from August 1, 2021 to July 31, 2022 and provided both blood samples and medical records. For patients with GD to be included in and excluded from the study, the following criteria were used. Inclusion criteria: (1) GD clinical diagnosis; (2) confirmed lack of acid beta-glucosidase activity according to an enzyme assay; (3) GD1 and GD3; and (4) confirmed molecular diagnosis of GD. Exclusion criteria: (1) subjects who passed away, declined to engage in the study, or could not be located; (2) subjects with medical or social issues that the researcher believed would prohibit compliance with the program or may raise the risk of involvement; (3) involvement in any ongoing clinical study; (4) existence of any additional life-threatening noncardiac condition; (5) hemoglobinopathies, chronic hemolytic anemias, portal hypertension brought on by liver conditions, and onco-hematological illnesses; (6) GD2 and (7) life-threatening cardiac condition. The Institutional Review Board and the Ethics Committee of the Children's Hospital of China Medical University approved the study (CMUH111-REC1-050). All participants, including the parents of participating children, signed a written informed consent form. The study corresponds to all relevant ethical standards. The characteristics of the patients in this study are recorded in Additional file 3. Further details and methods are available in the Additional file 4.

### Sample collection

We used BD Vacutainer ACD tubes (BD Biosciences) to collect blood samples from the six patients with GD and the six healthy donors (HDs). Within 4 h of collection, the samples were centrifuged, and the serum was decanted and stored at − 80 °C until use. Peripheral blood mononuclear cells (PBMCs) were isolated from samples collected in EDTA tubes from patients with GD and HDs using a gradient centrifugation procedure with Ficoll-Paque (GE Healthcare Life Sciences)[16]. To guarantee the high viability of isolated mononuclear cells, fresh blood was utilized. The sample was placed at the temperature between 18 and 20 °C. Whole blood was diluted with an equal volume of DPBS at room temperature, covered with Ficoll-Paque, and centrifuged (600×*g*, 30 min, and 20 °C) on an Allegra V-15R (Beckman) with acceleration at five and deceleration at zero (all other centrifugation steps were performed with acceleration at nine and deceleration at nine unless otherwise stated). The Ficoll-Paque–plasma interface was used to isolate PBMCs, which were then washed in RPMI-1640 medium containing 10% fetal calf serum (FCS) and resuspended in RPMI-FCS (500×*g*, 10 min, and 4 °C). Trypan blue and a Boeco hemocytometer were used to count and determine the vitality of PBMCs (Hamburg, Germany).

### DXA scanning

Whole-body DXA scans were performed using Hologic densitometers, adhering to the International Society for Clinical Densitometry (ISCD) guidelines for BMD assessment at specific skeletal locations [17, 18]. Since there are no standardized scores for Taiwan, we calculated the LS BMD Z-scores with DXA using the Han-Chinese children’s growth standard value and computed the height Z-scores [19]. According to the ISCD, measurement of pediatric BMD is indicated if a patient’s height Z-score is < − 1. The BMD Z-score was also adjusted for the height Z-score [20].

### Statistical analysis

We used GraphPad Prism 9 software (GraphPad Software, Inc.) for statistical analysis and data visualization. One-way analysis of variance was performed for comparisons among three groups, and pair-wise comparisons were performed using Tukey test. The Bonferroni correction was used to account for multiple comparisons and *P* value was set in 0.05 divided by total compared variants, *P* = 0.05/17 = 2.9 × 10^−3^). Pearson correlation coefficients were used to determine the association of NLRP3, caspase 1, and ASC protein levels in monocytes with osteoclast differentiation. All data were displayed as the mean ± standard error of the mean (SEM). *P* values of < 0.05 were considered statistically significant. Further details and methods are available in the Additional file 6.

## Results

### Analysis of the association of the *GBA1* gene and osteoporosis

**Fig. 1:**
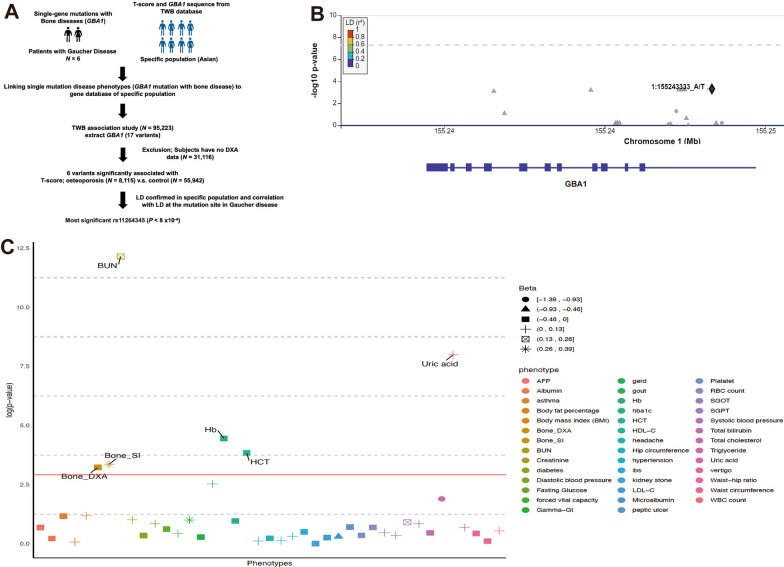
Identification of *GBA1* as an osteoporosis risk gene. **A** The study design overview presented here aims to investigate the relationship between the target gene and T-score from TWB. The pathway was designed based on the target gene summary statistics for T-score and a cosmopolitan linkage disequilibrium (LD) (Asian populations). The results from the cohorts were analyzed, using a Bonferroni-corrected *P* value threshold (0.05/17 = 2.94 × 10^−3^, where 17 is the number of analyzed *GBA1* alleles). **B** LocusZoom plots of *GBA1* gene (genotype and osteoporosis association, n = 64,107). The *Y* axis shows − log_10_(*P*) of association tests from *GBA1* gene. Highlighted variant is rs11264345 with top priority score with top association. **C** Manhattan plot of the PheWAS of pLOF variants in *GBA1* in TWB. The *X* axis displays phenotypes ordered by their *P* value within each disease group. The *Y* axis shows the strength of association, represented by the − log_10_(*P*) value, which was calculated using linear regression in the PheWAS model. The red line represents *P* = 0.05, while the red line represents Bonferroni's adjusted *P* = 0.05. *TWB* Taiwan BioBank, *LD* linkage disequilibrium, *DXA* dual-energy X-ray absorptiometry, *PheWAS* Phenome-wide association studies

To investigate the genetic factors that may increase the risk of osteoporosis, we conducted SNPs in the *GBA1* gene among individuals with Asian ancestry (N = 95,223; Table 1 and Fig. 1). The participants diagnosis with osteoporosis (T score ≤ − 2.5) were defined as case subjects (8,115 out of 64,057 individuals, 12.67% of the total cohort), while those without a diagnosed of osteoporosis (T score > − 1) were classified as controls (55,942 out of 64,057 individuals, making up 87.33% of the total cohort). The case group consisted of 58.4% and mean age of 56.93 ± 9.13 years, compared to the control group had 67% and a mean age of 47.30 ± 10.53 (*P* < 0.001; Table 1). Our analysis revealed six SNPs (rs9628662, rs2075569, rs1800442, rs3754485, rs1800438, and rs11264345) on chromosome 1 of the *GBA1* gene that were associated with BMD (all of 6 SNPs were passed the threshold of Bonferroni correction *P* = 0.05/17 = 2.9 × 10^−3^, Fig. 1A and Additional file 1). The most significant SNP, rs11264345, was located in the intronic region and had the largest effect on BMD (*P* < 8 × 10^−4^; Fig. 1B and Table 1). The locus rs11264345 was significantly different among males (*P* = 0.002; Table 1) but not among females (*P* = 0.143; Table 1).

**Table 1:** Demographics of the study population

	Overall (N = 64,057)			Male (N = 21,823)			Female (N = 42,234)		
	Normal (N = 55,942)	Osteoporosis (N = 8115)	*P* value	Normal (N = 18,444)	Osteoporosis (N = 3379)	*P* value	Normal (N = 37,498)	Osteoporosis (N = 4736)	*P* value
Age (year)	47.30 (10.53)	56.93 (9.13)	< 0.001*	48.07 (11.42)	54.46 (10.16)	< 0.001*	46.93 (10.04)	58.68 (7.86)	< 0.001*
Gender			< 0.001*			-			-
Male	18,444 (33.0%)	3379 (41.6%)		18,444 (100.0%)	3379 (100.0%)		0 (0.0%)	0 (0.0%)	
Female	37,498 (67.0%)	4736 (58.4%)		0 (0.0%)	0 (0.0%)		37,498 (100.0%)	4736 (100.0%)	
BMI (kg/m^2^)	24.38 (3.85)	23.65 (3.69)	< 0.001*	25.60 (3.54)	24.62 (3.64)	< 0.001*	23.78 (3.86)	22.95 (3.56)	< 0.001*
Smoking	14,387 (25.7%)	2464 (30.4%)	< 0.001*	10,186 (55.2%)	2153 (63.7%)	< 0.001*	4201 (11.2%)	311 (6.6%)	< 0.001*
Alcohol	2983 (5.3%)	533 (6.6%)	< 0.001*	2259 (12.3%)	462 (13.7%)	0.001*	724 (1.9%)	71 (1.5%)	0.100
Sport Habit	21,699 (38.8%)	3788 (46.7%)	< 0.001*	7986 (43.3%)	1444 (42.7%)	0.535	13,713 (36.6%)	2344 (49.5%)	< 0.001*
Menopause	13,341 (35.6%)	4172 (88.1%)	< 0.001*	–	–	–	13,341 (35.6%)	4172 (88.1%)	< 0.001*
eGFR^f^ (mL/min/1.73m^2^)	104.41 (14.6)	97.05 (15.01)	< 0.001*	97.83 (15.22)	94.68 (16.58)	< 0.001*	107.64 (13.12)	98.74 (13.53)	< 0.001*
rs11264345			0.001*			0.002*			0.143
TT	5027 (9.1%)	635 (7.9%)		1681 (9.2%)	247 (7.4%)		3346 (9.0%)	388 (8.3%)	
TA	22,979 (41.5%)	3302 (41.1%)		7561 (41.5%)	1371 (41.2%)		15,418 (41.5%)	1931 (41.1%)	
AA	27,411 (49.5%)	4097 (51.0%)		8987 (49.3%)	1713 (51.4%)		18,424 (49.5%)	2384 (50.7%)	

Values are presented as N (%) or mean (SD)

*eGFR* estimated glomerular filtration rate

*P value for chi square test or two independent t test

The age, BMI, smoking habits, exercise habits, menopausal status, and estimated glomerular filtration rate (eGFR) of the participants were significantly different in the case and control groups, regardless of their sex (Table 1). The results showed that all of these variables were significantly different between cases and controls, regardless of sex (Table 1). A multivariate logistic regression analysis was conducted to assess the impact of GBA1 on osteoporosis. The 8115 participants were tested for the presence of the risk allele (A) of rs11264345, with 51.0% found to be positive. The analysis was adjusted for various confounding factors, including age, sex, BMI, smoking habits, exercise habits, and eGFR. The results revealed that the presence of the risk allele was significantly associated with an increased risk of osteoporosis (Odds Ratio [OR] 1.06, 95% Confidence Interval [CI] 1.02–1.11; Table 2). However, a stratified analysis revealed that this locus was significantly different between cases and controls only in males. This finding suggests that the locus may play a role in the development of the osteoporosis in males (Table 2). The risk allele (A) of rs11264345 was present in 51.0% of the 8,115 participants and was associated with decreased bone mineral density (*P* < 8 × 10^−4^, OR 1.06, 95% CI 1.02–1.11; Table 2). Furthermore, the PheWAS analysis of a single variant, rs11264345 (the top variant identified by a genome-wide association study of osteoporosis with a *P* value of 8 × 10^−4^ in TWB), revealed significant associations with 5 health outcomes, including blood urea nitrogen (BUN), uric acid, hemoglobin (Hb), hematocrit (HCT), and bone stiffness index (Fig. 1C and Additional file 2). The risk allele of rs11264345 was found to exhibit a significant association with BUN and Uric acid in PheWAS (Fig. 1C). Previous research has linked the presence of splenomegaly, anemia, and thrombocytopenia, which are all significant clinical features of GD, are correlated with elevated levels of BUN and Uric acid [21–26]. Our findings underscore the role of *GBA1* in osteoporosis development and demonstrate the utility of PheWAS in investigating the roles of genes in complex diseases.

**Table 2:** Logistic regression results

	Overall (N = 64,057)			Male (N = 21,823)			Female (N = 42,234)		
	OR	95% CI	*P* value	OR	95% CI	*P* value	OR	95% CI	*P* value
Age (year)	1.12	(1.11, 1.12)	< 0.001*	1.07	(1.06, 1.07)	< 0.001*	1.13	(1.12, 1.14)	< 0.001*
Gender	0.66	(0.62, 0.71)	< 0.001*	–	–	–	–	–	–
BMI (kg/m^2^)	0.91	(0.91, 0.92)	< 0.001*	0.92	(0.91, 0.94)	< 0.001*	0.89	(0.88, 0.9)	< 0.001*
Smoking	1.16	(1.09, 1.24)	< 0.001*	1.34	(1.23, 1.45)	< 0.001*	–	–	–
Sport Habit	0.68	(0.64, 0.72)	< 0.001*	0.64	(0.59, 0.69)	< 0.001*	0.68	(0.63, 0.73)	< 0.001*
Menopause	–	–	–	–	–	–	2.87	(2.53, 3.26)	< 0.001*
eGFR (mL/min/1.73m^2^)	1.01	(1.01, 1.01)	< 0.001*	1.01	(1.01, 1.01)	< 0.001*	1.01	(1.01, 1.02)	< 0.001*
rs11264345	1.06	(1.02, 1.11)	0.002*	1.10	(1.04, 1.17)	0.002*	1.03	(0.98, 1.08)	0.270

*OR* odds ratio, *CI* confidence interval, *eGFR* estimated glomerular filtration rate

*Represent *P* value less than 0.05

### Exploring the linkage disequilibrium and correlation between rs11264345 and rs421016 (*GBA1*^L444P^) SNPs in patients with Gaucher Disease and their association with bone disease risk

We also investigated the linkage disequilibrium (LD) between two SNPs loci (rs11264345 from specific populations and rs421016, a rare variant from patients with GD, also known as GBA1 L444P mutation) in the GBA1 gene. Our results demonstrated potential linkage disequilibrium (LD) between the two loci, with a D’ value of 1, signifying that the variants are likely inherited together. However, the correlation degree between the two SNPs, measured by the r-squared value, was 0.0012, suggesting a low level of LD (Additional file 5) [27, 28]. In a sample of six GD patients, the majority (4 out of 6) presented with the rs421016 genotype and exhibited the rs11264345 locus in the form of the risk TA allele associated with bone diseases (Table 1 and Additional file 5). The remaining patients comprised one individual with missing data and another possessing the R120W/RecNciI genotype, which demonstrated the protective TT allele at the rs11264345 locus (Additional file 5). Based on this real-world evidence, there appears to be a correlation between the rs421016 and rs11264345 loci for the elevated risk of bone disease in patients with GD. Further studies are necessary to validate the present findings and fully elucidate the mechanisms underlying the association between GBA1 variants and osteoporosis.

### GBA1 mutation in monocytes from patients with GD significantly induces osteoclastogenesis

**Fig. 2:**
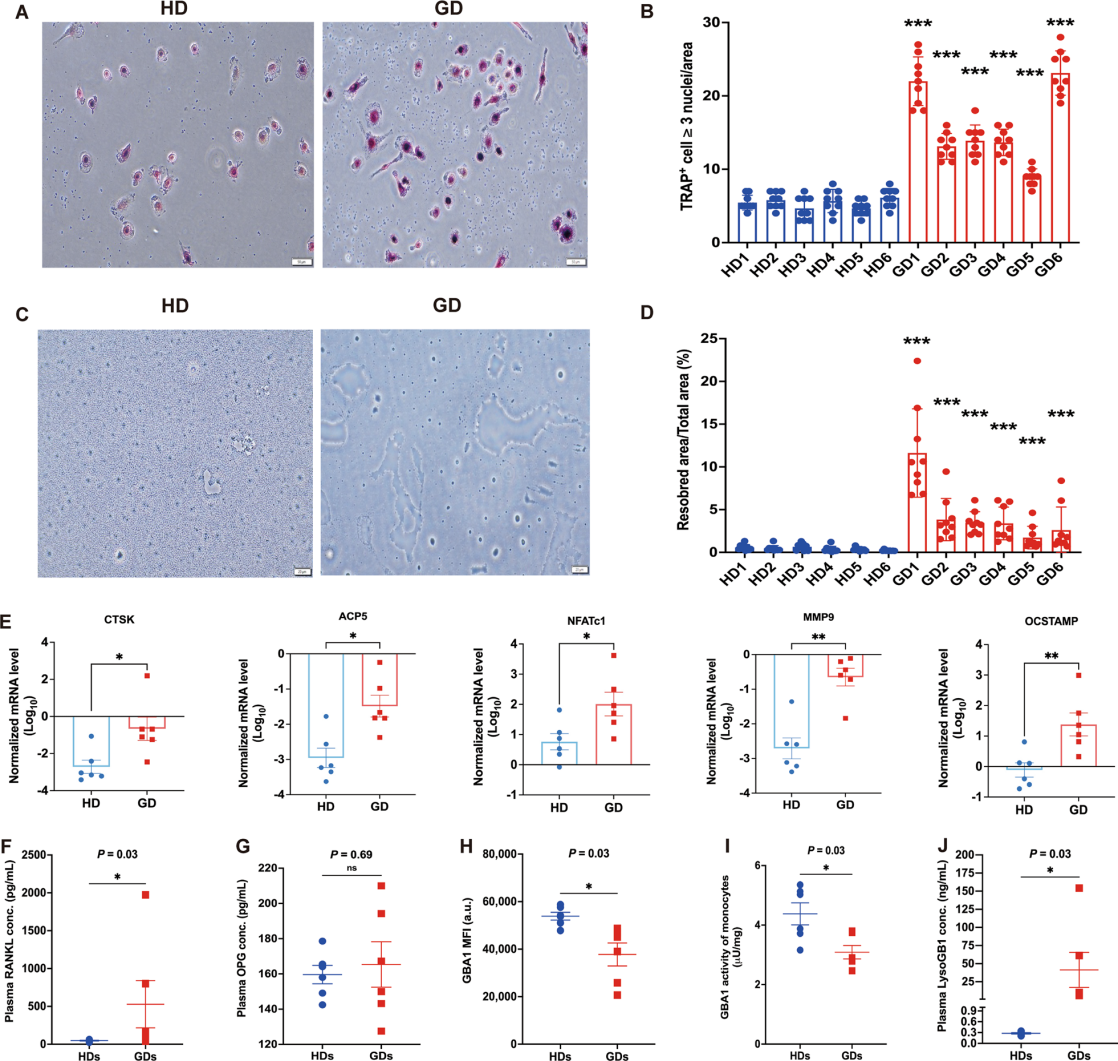
GBA1 mutation triggers osteoclastogenesis. **A** Representative images and **B** quantification of TRAP + polynucleated (≥ 3 nuclei) osteoclasts derived from PBMCs of patients with GD compared with age-, sex- and BMI-matched HDs (n = 6/group). Scale bar: 50 μm. **C** Representative images of the resorption assay. The blank area represents the location where osteoclasts have taken up material. **D** Percentage quantification of the resorbed area derived from PBMCs of patients with GD compared with age-, sex-, and BMI-matched HDs (n = 9/group). Scale bar: 20 μm. **E** Gene expression analysis of *CTSK*, *ACP5*, *NFATc1*, *MMP9*, and *OCSTAMP* in PBMCs of patients with GD compared with age-, sex- and BMI-matched HD osteoclasts (n = 6/group). **F** OPG and (G) RANKL was determined in plasma of patients with GD (n = 6) compared with age-, sex-, and BMI-matched HDs (n = 6). **H** Quantification of MFI of GBA1 in PBMCs of patients with GD compared with age-, sex-, and BMI-matched HDs (n = 6/group). **I** GCase activity was determined in PBMCs of patients with GD (n = 6) compared with age-, sex-, and BMI-matched HDs (n = 6). **J** Quantification of LysoGB1 concentration in plasma of patients with GD compared with age-, sex-, and BMI-matched HDs (n = 6/group). Data are presented as mean ± SEM. *P* values were determined by two-tailed paired Wilcoxon test (B and D-J). *P < 0.05, **P < 0.01, and ***P < 0.001

To further understand the pathophysiological significance of GBA1 mutation in monocytes undergoing osteoclast differentiation, we compared monocytes differentiated into osteoclast-like cells from patients with GD (n = 6) and from HDs (n = 6; Additional file 3). Monocytes from GD patients showed a higher proportion of mature tartrate-resistant acid phosphatase (TRAP)-positive multinucleated cells (indicative of osteoclast-like cells) compared to those from HDs (Fig. 2A). We assessed osteoclastogenesis by TRAP labeling at various induction times, defining osteoclasts as TRAP+ multinucleated cells with three or more nuclei. The quantitative results revealed that the induced monocytes from patients with GD had a significantly higher proportion of TRAP+ cells (*P* < 0.001 for HD1 vs. GD1, HD2 vs. GD2, HD3 vs. GD3, HD4 vs. GD4, HD5 vs. GD5, and HD6 vs. GD6; Fig. 2B). Moreover, GD patients exhibited a significantly increased resorption area and activity compared to HDs after various induction periods (Fig. 2C, D). Furthermore, the mRNA expression of osteoclast differentiation marker genes, such as Cathepsin K (*CTSK*), Acid phosphatase 5 (*ACP5,* also called *TRAP*), Nuclear factor of activated T cells 1 (*NFATc1*), Matrix metalloproteinase-9 (*MMP9*), and Osteoclast stimulatory transmembrane protein (*OCSTAMP*), was consistently and significantly increased in monocytes from patients with GD compared with HDs (*CTSK*, *P* = 0.03; *ACP5*, *P* = 0.02; *NFATc1*, *P* = 0.04; *MMP9*, *P* = 0.009; and *OCSTAMP*, *P* = 0.004; Fig. 2E). Together, these results imply that monocytes from patients with GD drive the functional dysregulation of osteoclast differentiation. We also quantified circulating bone turnover markers in plasma samples from participants with GD and matched control subjects using enzyme-linked immunosorbent assays (ELISAs). We observed significantly elevated levels of the osteoclastogenic factor receptor activator of nuclear factor kappa-Β ligand (RANKL) in patients with GD compared to controls (*P* = 0.03, Fig. 2F). However, there were no significant differences in concentrations of osteoprotegerin (OPG), a decoy receptor for RANKL, between the two groups (*P* = 0.69, Fig. 2G). Measurements of lyso-glycosphingolipid-1 (Lyso-GB1), a validated biomarker of GD, in plasma samples from patients and matched controls. As anticipated, concentrations of Lyso-GB1 were significantly elevated in GD patients compared to controls (*P* = 0.03, Fig. 2J). Monocyte’s GBA1 protein levels and enzymatic activities were concomitantly reduced in patients versus controls (*P* = 0.03, Fig. 2H; *P* = 0.03, Fig. 2I, respectively). Our results suggest that circulating monocytes in GD patients exhibit compromised GBA1 function and are predisposed to osteoclast differentiation, underscoring the complex interplay between GBA1 mutations and bone metabolism dysregulation in GD.

### ER expansion in monocytes from patients with GD

**Fig. 3:**
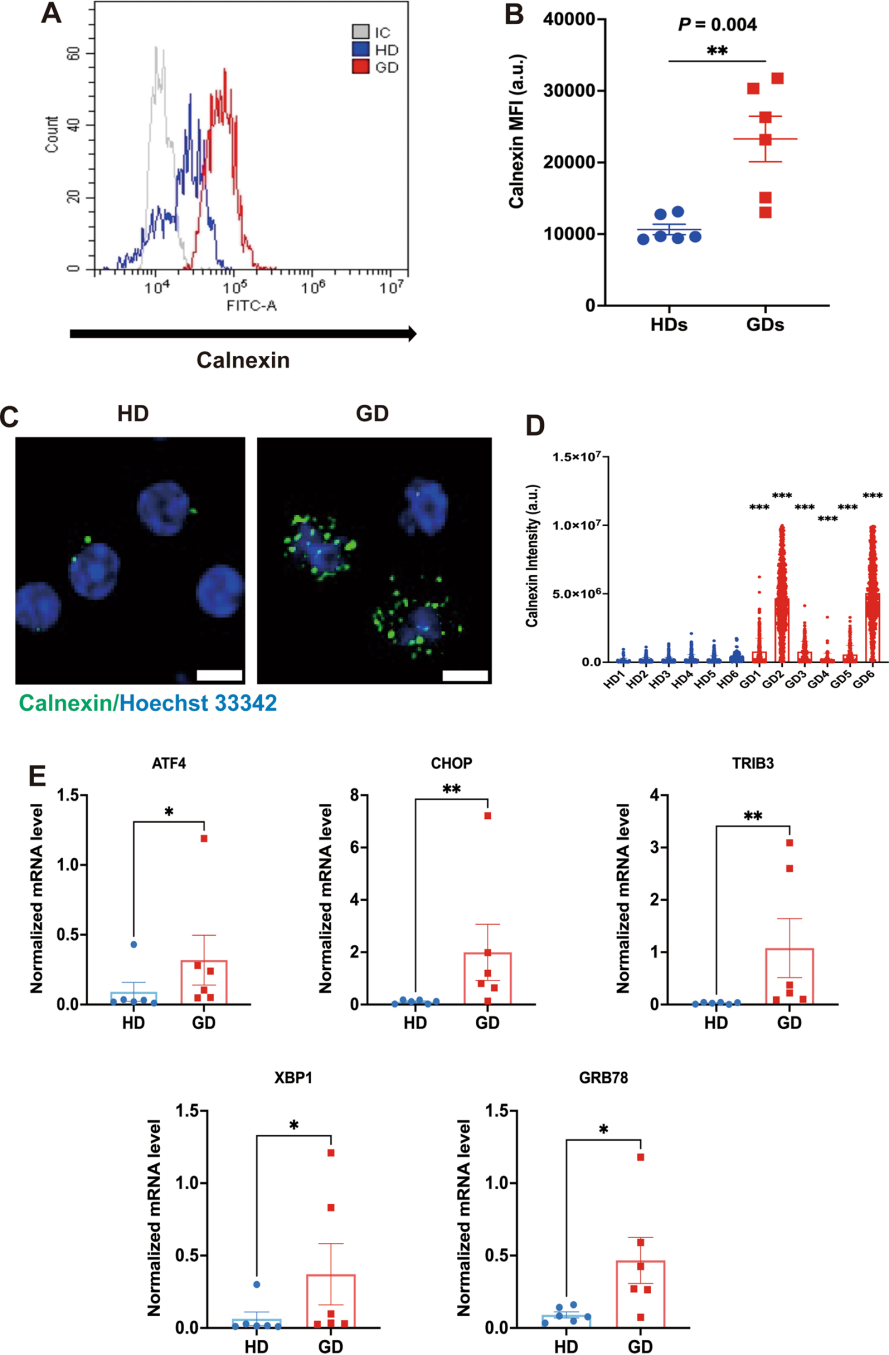
GBA1 mutation induces ER expansion and ER stress. **A** ER expansion in PBMCs of patients with GD. Histogram of calnexin (an ER marker) staining and overall median fluorescence intensity (MFI) in patients with GD compared with age-, gender- and BMI-matched HDs (n = 6/group). **B** Quantification of MFI of calnexin in PBMCs of patients with GD compared with age-, sex-, and BMI-matched HDs (n = 6/group). **C** Representative confocal microscopy imaging of calnexin in PBMCs of patients with GD compared with age-, sex-, and BMI-matched HDs. Green indicates calnexin, and blue indicates nuclei. Scale bar: 10 μm. **D** Quantification of MFI of calnexin in PBMCs of patients with GD compared with age-, sex-, and BMI-matched HDs. **E** Gene expression analysis of *ATF4*, *CHOP*, *TRIB3*, *XBP1,* and *GRP78* in PBMCs of patients with GD compared with age-, sex-, and BMI-matched HDs (n = 6/group). Data are presented as mean ± SEM. *P* values were determined by two-tailed paired Wilcoxon test (**B**, **D**, and **E**); *P < 0.05, **P < 0.01, and ***P < 0.001

Considering that ER biogenesis and expansion are believed to be influenced, at least in part, by the inflammatory signals associated with GD, we evaluated the presence of the ER expansion phenotype in monocytes from GD patients. We labeled calnexin, an ER marker, in monocytes from both HDs and patients with GD and calculated the median fluorescence intensity (MFI). Our findings revealed a significantly higher expression of calnexin in monocytes from GD patients (*P* = 0.004; Fig. 3A, B) hence confirming ER expansion. When compared with HDs, the MFI of calnexin in patients with GD showed an ER expansion and increased ER stress in the monocytes (Fig. 3C, D) and was significantly increased in monocytes from patients with GD (calnexin MFI, *P* < 0.001, Fig. 3D). Furthermore, the mRNA expression of ER stress marker genes, such as Activating transcription factor 4 (*ATF4*), C/EBP homologous protein (*CHOP*), Tribbles pseudokinase 3 (*TRIB3*), X-box binding protein 1 (*XBP1*), and Glucose-regulated protein-78 (*GRP78*), was significantly increased in monocytes of patients with GD compared with those of HDs (Fig. 3E). These findings imply that the accelerated bone crises and pathological bone disease found in a subgroup of patients with GD may be caused by the buildup of ER stress in monocytes.

### Inflammasome activation in monocytes from patients with GD

**Fig. 4:**
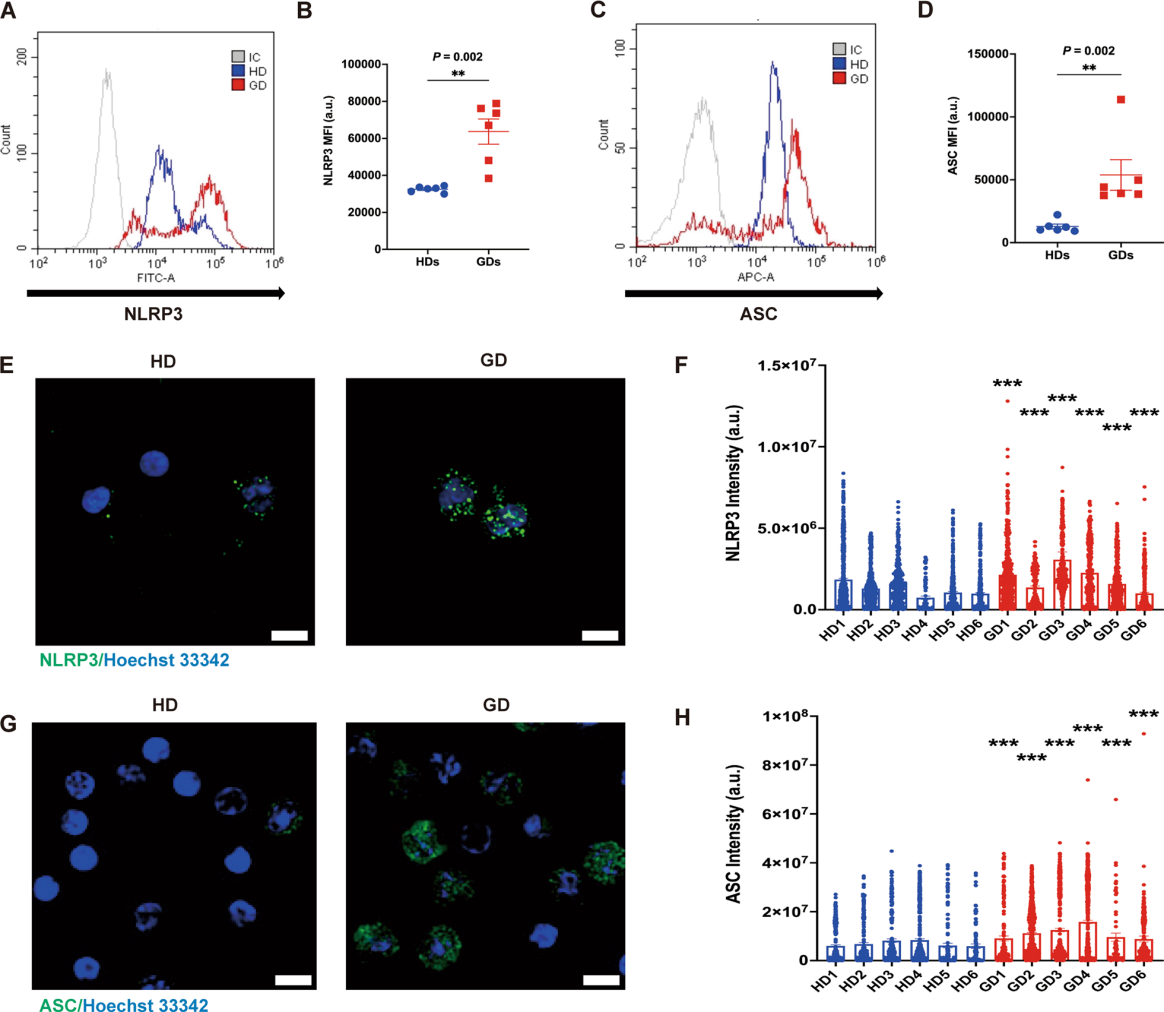
GBA1 mutation triggers Inflammasome activation in PBMCs of patients with GD. **A** NLRP3 and **C** ASC expression. **B** and **D** Quantification of the MFI of PBMCs of patients with GD compared with age-, sex-, and BMI-matched HDs (n = 6/group). Representative confocal microscopy imaging of **E** NLRP3 and **G** ASC in PBMCs of patients with GD compared with age-, sex-, and BMI-matched HDs. Green indicates NLRP3 or ASC, and blue indicates nuclei. Scale bar: 10 μm. Quantification of MFI of **F** NLRP3 and **H** ASC in PBMCs of patients with GD compared with age-, sex-, and BMI-matched HDs. Data are shown as mean ± SEM. *P* values were determined by two-tailed paired Wilcoxon test (**B** and **D**). *P < 0.05, **P < 0.01, and ***P < 0.001

Inflammasome activation is known to be induced by signals related to ER expansion [11]. To confirm the specific involvement of the NOD-, LRR- and pyrin domain-containing protein 3 (NLRP3) inflammasome, we analyzed NLRP3 and Apoptosis-associated speck-like protein containing a C-terminal caspase recruitment domain (ASC) in the monocytes of HDs and patients with GD using a flow cytometry and confocal microscopy. The fluorescence intensity of calnexin in patients with GD exhibited a signature shift compared with HDs (Fig. 4A, C). We observed that the levels of NLRP3 and ASC were significantly elevated in GD patients' monocytes, as demonstrated by the quantitative fluorescence intensity (*P* = 0.002, Fig. 4B; *P* = 0.002, Fig. 4D). These results indicate that the inflammasome is activated in the PBMCs of GD patients. By analyzing NLRP3 and ASC expression in monocytes from both HDs and GD patients and measuring the MFI, we confirmed that NLRP3, ASC expression and quantitative results were significantly higher in terms of expression in the monocytes of GD patients (Fig. 4E–H). Collectively, these results underscore the activation of the inflammasome in the monocytes of GD patients.

### Functional dysregulation of cytokines in patients with GD

**Fig. 5:**
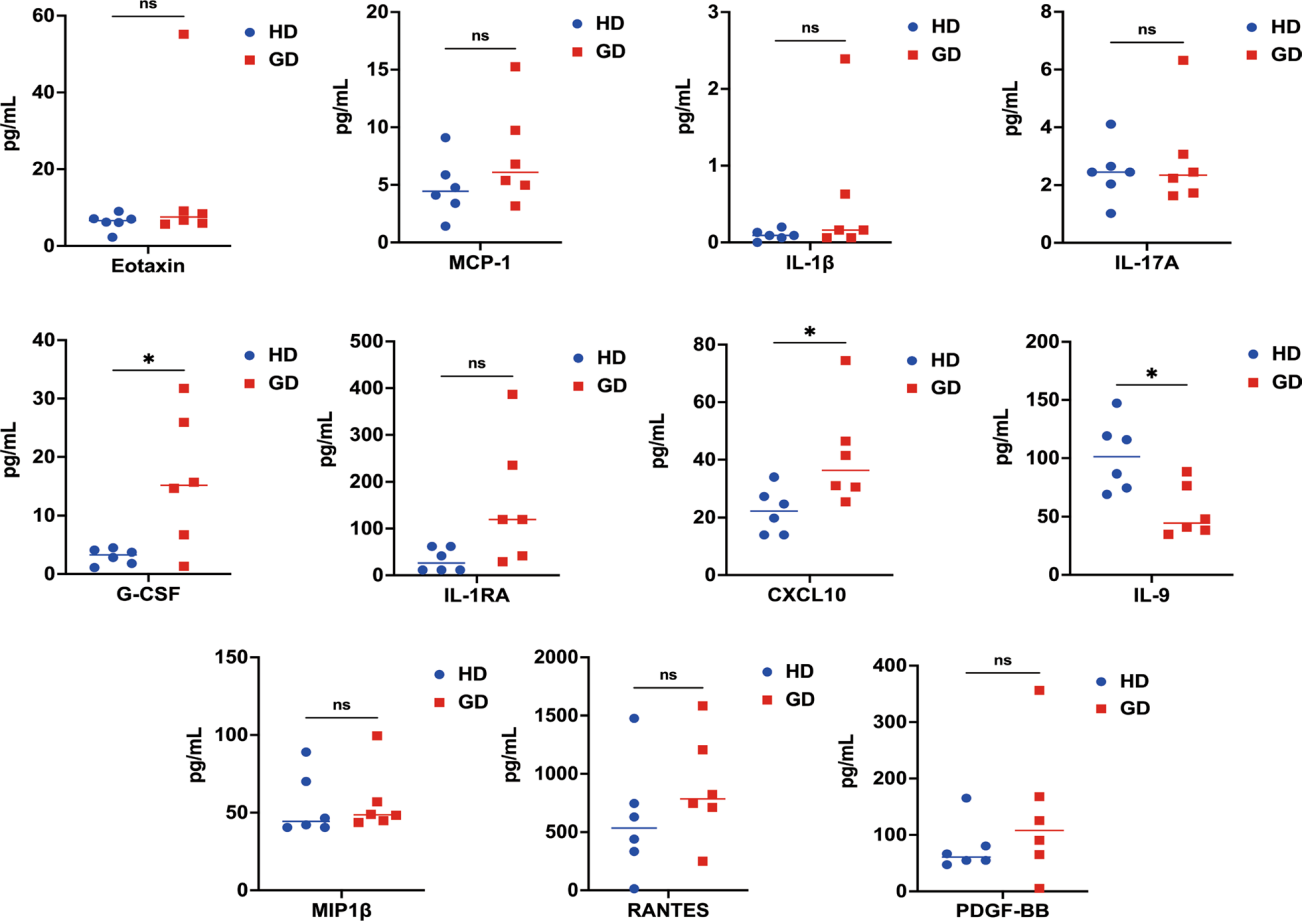
Functional dysregulation of plasma cytokines from patients with GD. Multiplex cytokine analysis of plasma from patients with GD (n = 6) compared with age-, sex-, and BMI-matched HDs (n = 6). Each dot represents an individual. The horizontal line represents the mean. G-CSF, *P* = 0.04; CXCL10, *P* = 0.03; and IL-9, *P* = 0.04 by two-tailed Mann–Whitney U-test

Cytokine dysregulation of has been implicated in the development of bone disease and osteoporosis [29]. To assess cytokines dysregulation, we identified the cytokines profiles in GD patients. Multiplex cytokine analysis revealed that significantly higher amounts of Granulocyte colony-stimulating factor (G-CSF, *P* < 0.05, Fig. 5) and CXC motif chemokine ligand 10 (CXCL10, *P* < 0.05, Fig. 5) are present in the plasma of patients with GD than the plasma of the HDs. The administration of G-CSF can affect the process of osteoblast formation in the bone marrow by inducing apoptosis of osteoblasts and concurrently increasing the number of osteoprogenitor cells [30]. The CXCL10 chemokine can indirectly stimulate osteoclastogenesis by enhancing the expression of RANKL and Tumour necrosis factor-α (TNF-α) in activated CD4^+^ T cells [31]. RANKL has also been found to induce the expression of CXCL10 in osteoclast precursors [31]. Additionally, we observed a significant reduction in the secretion of Interleukin-9 (IL-9, *P* < 0.05, Fig. 5). IL-9-producing type 2 innate lymphoid cells (ILC2s) are crucial for resolving chronic inflammation through molecular and cellular pathways [32]. In a mouse model, the absence of IL-9 was observed to result in impaired proliferation of ILC2s, activation of regulatory T cells (Treg), and chronic arthritis with excessive cartilage degradation and bone loss. Conversely, treatment with IL-9 has been shown to promote ILC2-mediated activation of Tregs and effectively resolve inflammation while protecting the bone [32]. No significant differences were detected for other cytokines, including eosinophil chemotactic protein (Eotaxin), Monocyte chemoattractant protein-1 (MCP-1), Interleukin-1β (IL-1β), Interleukin-17A (IL-17A), Interleukin-1 receptor antagonist (IL-1RA), Macrophage inflammatory protein-1β (MIP-1β), regulated on activation, normal T cell expressed and secreted (RANTES), and Platelet-derived growth factor-BB (PDGF-BB, Fig. 5). These findings suggested that the dysregulation of cytokines in patients with GD may play a critical role in inducing osteoclastogenesis.

### Ex vivo AAV9-GBA1 gene transfer into PBMCs of patients with GD

**Fig. 6:**
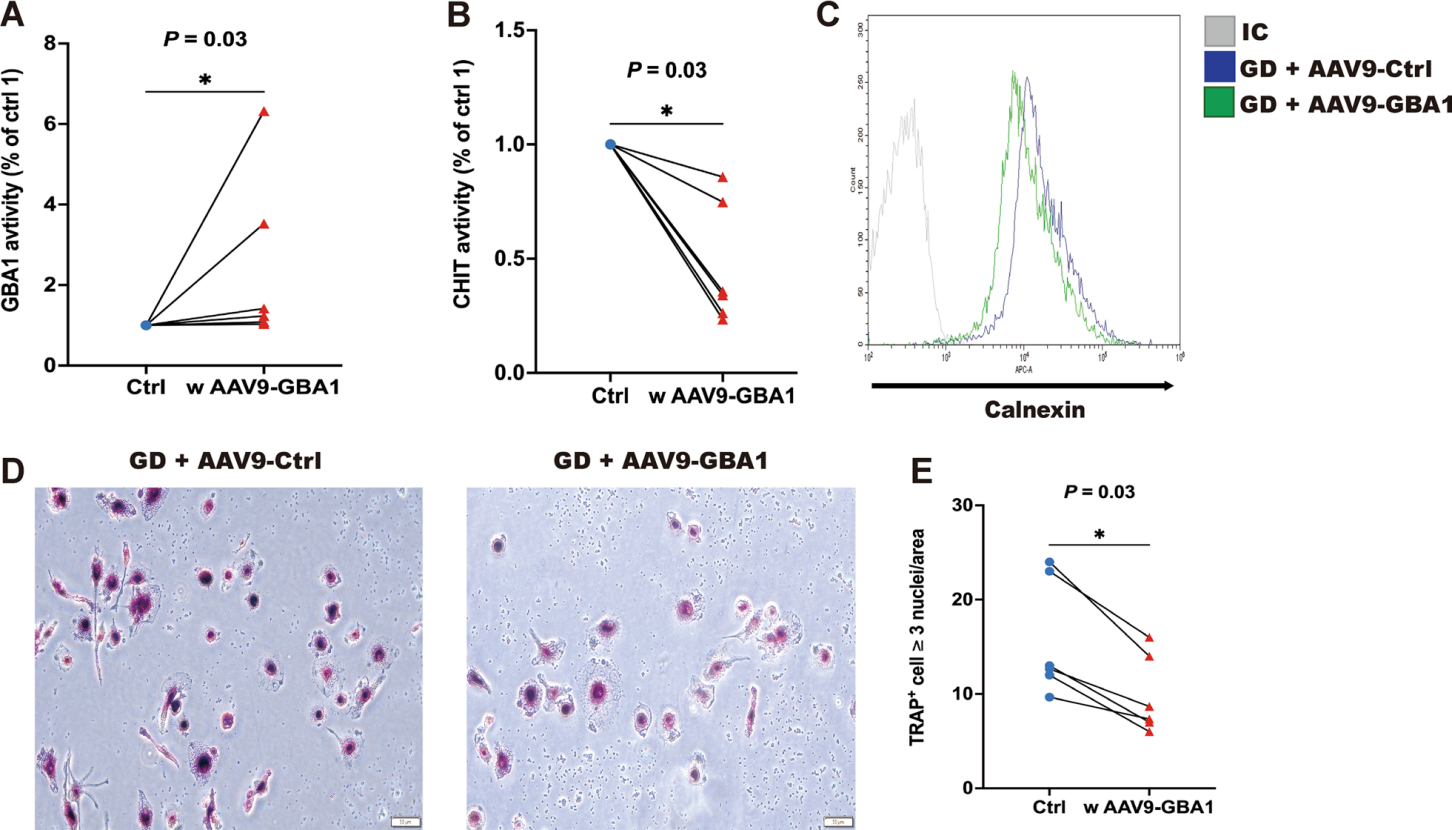
Reversal of GD symptoms by AAV9-GBA1 gene transfer. **A** GCase and **B** CHIT activity was determined in PBMCs of patients with GD (n = 6) compared with age-, sex-, and BMI-matched HDs (n = 6). **C** Representative histogram of the levels of ER chaperone protein calnexin measured by flow cytometry. PBMCs of patients with GD were treated with AAV9-Ctrl or AAV9-GBA1 (n = 3, independent experiments). Scale bar: 10 μm. **D** Representative images and **E** quantification of TRAP-positive polynucleated (≥ 3 nuclei) osteoclasts derived from PBMCs with GD treatment with AAV9-Ctrl or AAV9-GBA1 (n = 3 independent experiments). Transduction of AAV9-Ctrl or AAV9-GBA1 was for 3 days. *IC* isotype control

To assess whether functional GCase protein could be expressed in PBMCs from GD patients, we transfected these cells with plasmid AAV9-GBA1 or AAV9-Control (Ctrl) as shown in Fig. 6. We then utilized Western blot analysis with antibodies against GCase and actin, using actin as a loading control, to examine the cell lysates. The cells transfected with the AAV9-GBA1 plasmid produced supraphysiological amounts of GCase compared to the control cells, which likely expressed physiological levels of the protein. Quantification of relative protein expression revealed that GCase levels in the transfected cells were six times higher than in control cells. Further, a test for enzymatic activity confirmed that GCase from cells transfected with AAV9-GBA1 exhibited significantly greater activity (*P* = 0.03, Fig. 6A) than endogenous levels in control cells. Additionally, chitotriosidase (CHIT) activity, indicative of macrophage activation, decreased in PBMCs from GD patients following transduction with AAV9-GBA1, compared to control cells (*P* = 0.03, Fig. 6B).

We used an ex vivo model with AAV9-Ctrl or AAV9-GBA1 to examine whether ER enlargement and osteoclast activation were reversed in response to AAV9-GBA1 therapy. Our results revealed that the ER marker calnexin was decreased in PBMCs of patients with GD transfected with AAV9-GBA1 compared with the control cells (Fig. 6C). Similarly, the number of osteoclast-like cells decreased (Fig. 6D). After treatment with or without AAV9-GBA1, we detected osteoclast-like cells via TRAP labeling. The presence of TRAP-positive cells indicated osteoclast differentiation, with osteoclasts defined as TRAP+ cells containing three or more nuclei. Quantitative analysis revealed a significantly lower proportion of TRAP^+^ cells differentiated from monocytes of GD patients in response to AAV9-GBA1 treatment compared to controls (*P* = 0.03, Fig. 6E). Collectively, these data showed that ER expansion due to decreased GCase activity in GD is also reversed with AAV9-GBA1 therapy.

### BMD in patients with GD

**Fig. 7:**
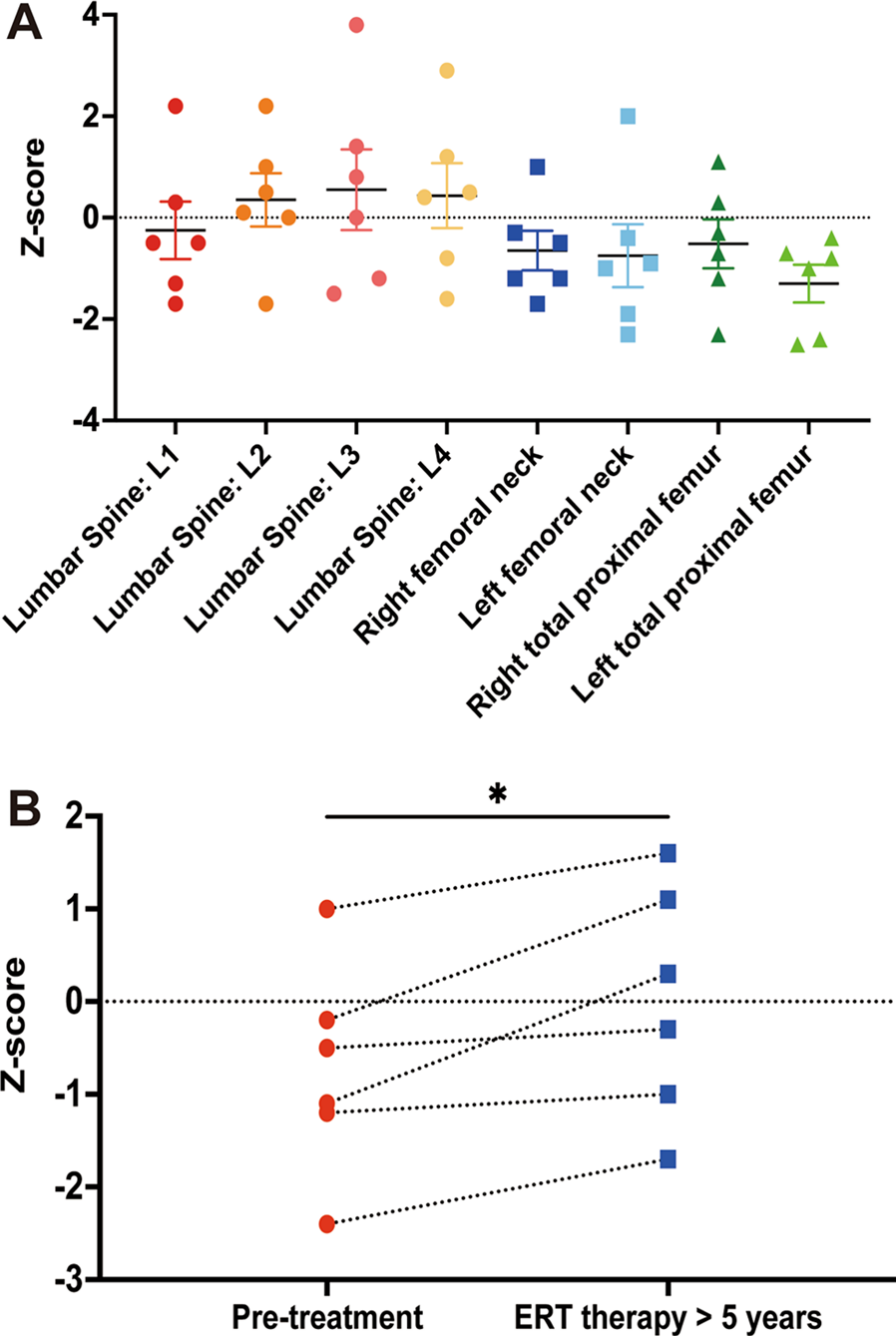
BMD data of patients with GD. **A** Scatter plots of the Z scores of LS (L1–L4), femoral neck, and total hip BMD in patients with GD. Data are shown as means ± SEM. **B** Z scores of the LS (mean of L1–L4) from patients with GD that underwent ERT therapy. Each dot represents an individual (n = 6)

DXA data were obtained for all study participants. The mean LS BMD Z-scores were as follows: − 0.25 ± 0.57 in L1, 0.35 ± 0.52 in L2, 0.55 ± 0.79 in L3, and 0.43 ± 0.64 in L4. Additionally, the mean BMD Z-score was − 0.65 ± 0.39 in the right femoral neck, − 0.75 ± 0.62 in the left femoral neck, − 0.52 ± 0.48 in the right total proximal femur, and − 1.3 ± 0.37 in the left total proximal femur (Fig. 7A). According to the 2019 ISCD standard, 50% (3 out of 6) of the patients were diagnosed with BMD below the expected range for their age (Z-score: <  − 2), while the BMD of the remaining GD patients was within the normal range. Half of the patients initiated ERT within 1–3 years following their diagnosis, whereas the other half started ERT between the ages of 13 and 18. The patients who began ERT early, under 5 years of age, had their DXA values indicating only the mean values for LS L1–L4. Significantly lower LS L1–L4 BMD Z-scores were observed in GD patients after undergoing ERT (*P* = 0.03, Fig. 7B). These data show that the GD patients who start ERT early maintain normal BMD levels.

## Discussion

Our study has clarified the association between GBA1 variant and bone diseases across both rare and common conditions. Through the analysis of T-scores and GBA1 sequences, we identified variants that decrease bone mineral density, thereby increasing the risk for osteoporosis and fractures. Our results underscore the critical roles of ER stress and inflammasome activation in the differentiation of GBA1-deficient monocyte osteoclasts, shedding light on the mechanisms that underpin bone disorders associated with GBA1 mutations.

Analysis of genetic and bone mineral density data on over 64,000 individuals revealed that the GBA1 single nucleotide polymorphism rs11264345 is significantly associated with increased risk of osteoporosis (OR 1.06, *P* < 0.002), indicating this gene variant promotes bone density loss at a population level. An OR of 1.06 suggests this SNP confers only minimal elevated susceptibility for reduced bone mineral density, though the finding was statistically significant. Further investigation showed 4 out of 6 GD patients possessing a rare GBA1 mutation (L444P) also carried the risk variant of rs11264345, evidencing a possible correlation between GBA1 mutation (L444P) and common variants elevating osteoporosis susceptibility. These integrated genotypic findings link compromised glucocerebrosidase enzymatic activity to heightened predisposition and accelerated development of skeletal fragility across rare and common conditions.

The 6 GD cases displayed heterogeneity in age at diagnosis (2–40 years), GBA1 genotypes (4 L444P/L444P, 1 R120W/RecNciI, 1 N227S/L444P), enzyme therapy duration (14–23 years) and organ involvement. All patients had hepatomegaly; splenomegaly occurred in R120W/RecNciI and N227S/L444P variants. Notably, greater bone density preservation was observed for patients treated earlier following diagnosis irrespective of mutation. While serum calcium, phosphorus and alkaline phosphatase mostly remained in normal ranges, abnormalities emerged in mineral metabolism for specific genotypes (reduced calcium for R120W/RecNciI and elevated phosphorus for N227S/L444P).

Bone involvement is prevalent among GD patients, manifesting even in asymptomatic cases. Symptoms range from decreased bone mineral density and osteonecrosis to fractures, bone pain, and crises. It has been shown that RANKL-induced ER stress can affect osteoclast differentiation by activating the cAMP-response element-binding protein H (CREBH). Furthermore, the NLRP3 inflammasome interacts with damaged mitochondria, stimulating inflammation [33, 34]. The NLRP3 inflammasome's role may be pivotal in pathological osteolysis, it seems to be less involved in baseline bone remodeling [35]. The upregulation of ER stress genes during osteoclastogenesis underscores the strong connection between osteoclastogenesis, ER stress, and inflammasome activation [34].

Accumulation of Lyso-GB1 has been shown to inhibit protein kinase C across various cell types, suggesting therapeutic targets beyond macrophages [36]. Additional research found sphingolipids drive aberrant osteoclast expansion and dysfunctional osteoblasts, creating an imbalance in bone remodeling. The activation of NLRP3 inflammasome and the diversion of mesenchymal stem cells (MSCs) towards adipocytes are also believed to contribute to GD-related bone loss [37]. Reed et al. investigated the direct effects of glucosylceramide and glucosylsphingosine on MSCs, osteoblasts, osteoclasts and plasma cells in GD [37]. Also, the phenotype of plasma cells was used to connect the symptoms between patients with multiple myeloma and GD [37]. They hypothesized that excess levels of these sphingolipids drive aberrant expansion of osteoclasts coupled with dysfunctional osteoblasts, creating an imbalance in bone remodeling that culminates in osteoporosis [37]. Crivaro et al. examined how impaired differentiation of MSCs into osteoblasts accelerates osteoclastogenesis in GD [38], identifying the activation of the NLRP3 inflammasome and the shift of MSCs towards an adipogenic phenotype as key factors driving bone marrow loss in GD patients [38]. Separately, our group identified a GBA1 variant increasing osteoporosis risk and found it to be associated with severe skeletal disease phenotypes, thereby linking the GBA1 genotype to bone pathology and highlighting the genetic determinants of heterogeneity.

Accumulation of glucosylceramide in GD may disrupts androgen signaling by altering the composition of cell membrane lipid rafts, which leads to reducing bone formation [39–41]. Androgens are essential for maintaining bone density [41], and impaired androgen receptor signaling can accelerates bone loss, with greater effects in males [41]. Glucosylceramide also causes dysregulation of osteoblast and osteoclast signaling [37]. This explains accelerated bone loss and differences between sexes in GD patients.

The 6 GD cases exhibited heterogeneity in age at diagnosis, ranging from 2 to 40 years old. Of the cases, 3 out of 6 had below expected BMD Z-scores, indicating below average BMD for age, while the remaining 3 had Z-scores within the normal range. It is noted that 50% of patients initiated ERT within 1–3 years following initial Gaucher diagnosis. In contrast, the other 50% began ERT treatment later, between the ages of 13–18 years. Upon examination, the 50% of cases with normal BMD Z-scores were the younger subgroup who had received ERT shortly after diagnosis, within 1–3 years. The 3 cases with BMD scores below expectation were from the later treatment group, having started therapy during adolescence between ages 13–18. In summary, of the 6 GD patients, those receiving earlier ERT following diagnosis demonstrated preservation of normal age-adjusted bone density, whereas cases with delayed treatment initiation exhibited below average BMD relative to peers. However, given the small sample size, further studies are required to conclusively determine if age at ERT start significantly impacts bone density in GD.

The results of this study suggest several implications for medical practice. Firstly, genetic screening for GBA1 variants may prove useful for identifying patients at higher inherent risk for osteoporosis, enabling earlier monitoring and preventative interventions before bone loss becomes severe. Additionally, closer monitoring of bone density in GD could facilitate earlier diagnosis and treatment, particularly in patients presenting with isolated skeletal symptoms without hepatosplenomegaly or cytopenia. Our findings indicate starting enzyme therapy sooner after GD diagnosis confers better outcomes for preserving bone integrity. Finally, the correlations found between monocyte pathology and bone disease activity support using circulating monocyte analyses to gauge skeletal complications and response to therapy in GD patients.

Our limitations including lack mouse models and small sample sizes must be addressed through larger, controlled studies correlating genotypes and clinical disease. Though this study provides novel insights, a key limitation is the relatively small number of GD patients included (n = 6). This restricts the generalizability of findings to broader GD populations with heterogenous disease phenotypes and genotypes. Further studies with more GD patients are warranted to validate if the identified association holds in larger cohorts and determines the clinical utility of genotyping these loci to guide management for skeletal complications in GD. The need for a transgenic mouse model to explore how early cell lines and molecular regulatory mechanisms are involved in bone disease progression. Elucidating these relationships can optimize management across GD patients.

Delayed GD diagnosis often results in more severe bone disease. Since bone complaints are frequently the initial symptoms, diagnosis can be prolonged in the absence of visceromegaly or hematologic abnormalities. Early intervention prevents irreversible bone complications. Our results discovered links between monocyte levels and bone symptoms, emphasizing closely monitoring bone health to minimize progression, especially with isolated bone involvement. Early administration of ERT may significantly benefit such patients.

## Conclusions

This study demonstrates that variants in the GBA1 gene constitute a genetic risk factor predisposing specific populations to osteoporosis, as evidenced by the significant association between the GBA1 variant rs11264345 and reduced bone mineral density. The risk allele of this variant was present in 51% of osteoporosis cases and conferred increased odds of bone disease. Longitudinal bone density data indicates that initiating enzyme replacement therapy earlier following Gaucher diagnosis leads to greater preservation of bone mineral density over time. This suggests a possible window for effectively minimizing skeletal complications through early treatment.

In summary, characteristic GBA1 variants predispose to low bone density in the general population. In Gaucher disease, loss of glucocerebrosidase function triggers downstream inflammation and osteoclast activity culminating in skeletal fragility. Elucidating mechanisms connecting genotype to bone disease phenotypes can guide management to improve quality of life for these patients.

### Supplementary Information

**Additional file 1:** List of the 17 Osteoporosis-associated GBA1 SNPs genotyped in the study cohort.

**Additional file 2:** pheWAS analysis.

**Additional file 3:** Characteristics of the patients with Gaucher disease in this study.

**Additional file 4:** Supplementary Methods.

**Additional file 5:** Linkage equilibrium between rs11264345 and rs421016.

**Additional file 6:** The primer information for quantitative PCR.

## Data Availability

Information related to patient privacy is not available, other data are available on reasonable request.

## Abbreviations

ASC: Apoptosis-associated speck-like protein containing a caspase recruitment domain
AVN: Avascular necrosis
BMD: Bone mineral density
BI: Bone infarct
CHI: Chitotriosidase
CREBH: CAMP-responsive element binding protein, hepatocyte specific
CXCL10: C-X-C motif chemokine ligand 10
DXA: Dual-energy X-ray absorptiometry
ER: Endoplasmic reticulum
ERT: Enzyme replacement therapy
GCase: Glucocerebrosidase
GD: Gaucher disease
HD: Healthy donor
IL: Interleukin
ISCD: International Society for Clinical Densitometry
LS: Lumbar spine
M-CSF: Macrophage colony-stimulating factor
MFI: Median fluorescence intensity
NLRP3: NLR family pyrin domain containing 3
PBMC: Peripheral blood mononuclear cell
RANKL: Receptor activator of nuclear factor-κB ligand
TRAP: Tartrate-resistant acid phosphatase
