# Supplementary material for: GBA1 as a risk gene for osteoporosis in the specific populations and its role in the development of Gaucher disease
Source: Orphanet J Rare Dis. 2024 Apr 4;19:144. doi: 10.1186/s13023-024-03132-x (PMC10993575; doi:10.1186/s13023-024-03132-x)
Supplement: Supplementary file 1 — Additional file 1: List of the 17 Osteoporosis-associated GBA1 SNPs genotyped in the study cohort. [file 13023_2024_3132_MOESM1_ESM.docx]

Additional file 1. List of the 17 Osteoporosis-associated GBA1 SNPs genotyped in the study cohort.

| **rsID** | **Gene** | **CHR** | **pb38** | **A1** | **A2** | **Genotype frequency Case** | **Genotype frequency Control** | ***P*** |
| --- | --- | --- | --- | --- | --- | --- | --- | --- |
| rs9628662 | GBA1 | 1 | 155236550 | T | G | 639/3321/4100 | 5050/23048/27466 | 0.001079 |
| rs572653386 | GBA1 | 1 | 155236882 | T | C | 0/9/8088 | 0/32/55757 | 0.074 |
| rs2075569 | GBA1 | 1 | 155239569 | C | T | 632/3303/4095 | 5007/22933/27414 | 0.0008742 |
| rs142348200 | GBA1 | 1 | 155240336 | T | C | 5/267/7785 | 16/1942/53653 | 0.2205 |
| rs146532106 | GBA1 | 1 | 155240394 | C | G | 11/687/7351 | 118/4770/50640 | 0.3622 |
| rs139190127 | GBA1 | 1 | 155240461 | C | T | 0/31/8078 | 0/193/55709 | 0.598 |
| rs567244045 | GBA1 | 1 | 155241974 | T | C | 0/38/8051 | 1/250/55499 | 0.897 |
| rs12041778 | GBA1 | 1 | 155242046 | T | C | 0/57/7930 | 1/412/54800 | 0.885 |
| rs141571262 | GBA1 | 1 | 155242225 | T | G | 0/5/8094 | 0/85/55727 | 0.042 |
| rs1800442 | GBA1 | 1 | 155242309 | A | G | 635/3305/4095 | 5023/22980/27410 | 0.0009155 |
| rs3754485 | GBA1 | 1 | 155242420 | A | G | 633/3301/4096 | 5011/22944/27412 | 0.0008692 |
| rs1800438 | GBA1 | 1 | 155242497 | C | T | 635/3305/4096 | 5024/22981/27411 | 0.0008807 |
| rs888479781 | GBA1 | 1 | 155242540 | T | C | 0/13/8090 | 0/62/55820 | 0.224 |
| rs188491571 | GBA1 | 1 | 155242597 | A | C | 0/12/8078 | 0/84/55693 | 0.961 |
| rs11264345 | GBA1 | 1 | 155243333 | T | A | 635/3302/4097 | 5027/22979/27411 | 0.0008008 |
| rs187014985 | GBA1 | 1 | 155243467 | A | T | 5/267/7785 | 16/1942/53652 | 0.2205 |
| rs989181972 | GBA1 | 1 | 155243643 | C | T | 0/5/8080 | 0/45/55675 | 0.57 |

SNP (single nucleotide polymorphism); CHR (chromosome); A1 (allele 1); A2 (allele 2)
