# Supplementary material for: GBA1 as a risk gene for osteoporosis in the specific populations and its role in the development of Gaucher disease
Source: Orphanet J Rare Dis. 2024 Apr 4;19:144. doi: 10.1186/s13023-024-03132-x (PMC10993575; doi:10.1186/s13023-024-03132-x)
Supplement: Supplementary file 3 — Additional file 3: Characteristics of the patients with Gaucher disease in this study. [file 13023_2024_3132_MOESM3_ESM.docx]

Additional file 3. Characteristics of the patients with Gaucher disease in this study.

| Patients number | 1 | 2 | 3 | 4 | 5 | 6 |
| --- | --- | --- | --- | --- | --- | --- |
| Gender | F | M | M | M | F | M |
| Age (years) | 20 | 34 | 34 | 17 | 19 | 70 |
| Gaucher type | 1 | 1 | 1 | 1 | 3 | 3 |
| Age at Onset (years) | 3 | 18 | 2 | 3 | 3 | 40 |
| Genotype | L444P/L444P | L444P/L444P | L444P/L444P | R120W/Rec*Nci*I | L444P/L444P | N227S/L444P |
| Enzyme replacement treatment duration (years) | 16 | 16 | 21 | 14 | 16 | 23 |
| Liver |  |  |  |  |  |  |
| GOT (AST) (U/L, <34) | /16 | /25 | /27 | 37/19 | /31 | /24 |
| GPT (ALT) (U/L, <40) | /10 | /17 | /21 | 10/20 | /15 | /24 |
| Hepatomegaly | Liver parenchymal disease | Liver parenchymal disease | Liver parenchymal disease / small liver | Liver parenchymal disease | Liver parenchymal disease | Liver nodule  Liver score 7  + |
| Spleen-Splenomegaly | Normal | Normal | Absence / splenectomy | + | Normal (improved) | splenectomy |
| Neurologic | tremor |  |  |  | seizure | seizure |
| Thrombocytopenia |  | Normal (improved) | Normal |  |  |  |
| Anemia | Hb: 11.5 | Hb: 15.6 | Hb: 14.5 | Hb: 10.7  Thalassemia type A Regular transfusion | Hb: 13.7 | Hb: 14.5 |
| Pancytopenia | - | - | - | - | - | - |
