# Supplementary material for: GBA1 as a risk gene for osteoporosis in the specific populations and its role in the development of Gaucher disease
Source: Orphanet J Rare Dis. 2024 Apr 4;19:144. doi: 10.1186/s13023-024-03132-x (PMC10993575; doi:10.1186/s13023-024-03132-x)
Supplement: Supplementary file 4 — Additional file 4: Supplementary Methods. [file 13023_2024_3132_MOESM4_ESM.docx]

Additional file 4

Method

**Flow cytometry and monocyte isolation**

Flow cytometric cell sorting (FACSAria III cell sorter; BD Biosciences; ≥95% purity) was used to purify CD14+ monocytes from PBMCs. We cultured monocytes in RPMI-1640 complete medium with 10% heat-inactivated human AB serum (Sigma, H3667) at 37°C in a 5.5% CO_2_ environment for 2 days. Measurement of CD14 in monocytes to measure purification ratio using multiparameter flow cytometry and the following antibody: fluorescein isothiocyanate-labeled anti-human CD14 antibody (BioLegend, clone: M5E2, #301804, [RRID: AB_314186](https://antibodyregistry.org/search.php?q=AB_314186), 1:100).

**Multiplex cytokine assay**

All serum samples were stored at −80°C. Multiplex tests were performed as follows. The 27-plex human cytokine panel, which assesses for FGF basic, Eosinophil chemotactic protein (Eotaxin-1), Granulocyte-colony stimulating factor (G-CSF), Granulocyte macrophage-colony stimulating factor (GM-CSF), Interferon-gamma (IFN-γ), Interleukin-1 beta (IL-1β), IL-1ra, IL-2, IL-4, IL-5, IL-6, IL-7, IL-8, IL-9, IL-10, IL-12 (p70), IL-13, IL-15, IL-17, Interferon gamma-induced protein 10 (IP-10, also known as C-X-C motif chemokine ligand 10, CXCL10), Monocyte chemoattractant protein-1 (MCP-1), Macrophage Inflammatory Proteins-1 alpha (MIP-1α), Macrophage Inflammatory Proteins-1 beta (MIP-1β), Platelet-derived growth factor-BB (PDGF-BB), Regulated upon Activation, Normal T Cell Expressed and Presumably Secreted (RANTES), Tumor Necrosis Factor-alpha (TNF-alpha), and Vascular endothelial growth factor (VEGF) (Bio-Plex Pro 27-plex; Bio-Rad) was used according to the manufacturer’s instructions. The assays were performed using an automated magnetic wash station (Bio-Plex Pro II; Bio-Rad) for the washing procedures. The Luminex® 200TM system (Bio-Rad) was used to perform the tests and gather fluorescence results. Using Bio-Plex manager software (version 5.0; Bio-Rad), the calibration curves for each cytokine were analyzed using five parametric logistic curve regressions. When the points fell between 80% and 120% of the predicted values, standard values were deemed acceptable. The resulting data were examined using GraphPad Prism (version 9.0.1; USA).

**Preparation of rAAV9 vectors**

All rAAV9 vectors utilized in this study were prepared by the Genetics and Metabolism Lab at the Children’s Hospital of China Medical University, Taichung, Taiwan. A trans plasmid containing the Rep gene (AAV2), Cap gene (AAV9), an adeno helper plasmid, and a cis plasmid containing the rAAV-containing transgene cassette and inverted terminal repeats (AAV2) were triple-transfected into HEK293 cells to create all rAAV vectors. The isolated AAV9-Control (Ctrl, empty backbone vector) and AAV9-GBA1 vectors were purified by dialysis and cesium chloride gradient centrifugation. We used a Taqman quantitative polymerase chain reaction (qPCR) assay for AAV titration to determine the rAAV genome copy number(20).

**Osteoclast differentiation and tartrate-resistant acid phosphatase (TRAP) staining**

We added the isolated PBMCs to 96-well plates (for the TRAP assay; 3 × 10^5^/well) and 48-well plates (for the RNA assay; 7.5 × 10^5^/well) containing 100 μL/well or 200 μL/well, respectively, of RPMI-1640 medium (10% FCS and 1% penicillin/streptomycin) and incubated them for 1–2 h at 37°C using plastic adhesion to filter monocytes in a 5.5% CO_2_ environment. The cells were then rinsed, and RPMI-1640 medium (1% penicillin/streptomycin and 10% FCS) was used to differentiate the cells into osteoclasts with 20 ng/mL macrophage colony-stimulating factor (M-CSF), 50 ng/mL Receptor activator of nuclear factor-κB ligand (RANKL), and 10 ng/mL transforming growth factor β (all PeproTech) at 37°C in an atmosphere of 5.5% CO_2_. On days 3 and 5 of differentiation, two-thirds of the medium was changed. After 5 days, the preosteoclasts were incubated as indicated with Ctrl or GBA1 protein, generated by four freeze/thaw cycles of the remaining PBMCs. We examined the osteoclasts using tartrate-resistant acid phosphatase (TRAP) staining after they had differentiated (day 10). We acquired images using a microscope (CKX53, Olympus) and cellsens software (Olympus). The osteoclasts were quantified using ImageJ. To identify osteoclasts, differentiated osteoclast-like cells were fixed in 4% paraformaldehyde. The number of TRAP^+^ multinucleated cells (≥3 nuclei) per unit area was determined by one author (KWL) who was blinded to the treatments.

**Resorption assay**

We conducted a resorption test by culturing 1 × 10^6^ monocytes per well in 24-well plates covered with calcium phosphate (Corning) and containing 1 mL of osteoclast medium with 20 ng/mL M-CSF and 50 ng/mL RANKL at 37°C in a 5.5% CO_2_ environment. Every two days, the medium was changed. We lysed the osteoclasts with ddH_2_O after differentiation had taken place for 5 days. The plates were then rinsed with ddH2O, treated for 5 min with 5% sodium hypochlorite (Millipore-Sigma), and dried for 2 h at room temperature. A microscope (CKX53, Olympus) and cellsens software (Olympus) were used to capture images. The osteoclasts were quantified, and the percentage of the resorbed area was determined using ImageJ.

**RNA extraction and qPCR**

Utilizing TRIzol reagent, total RNA was isolated from monocytes (Sigma, T9424). RNase-free DNase (Thermo Fisher, EN0521) was used to treat the RNAs in human samples before the isolated RNA underwent reverse transcription using a High-Capacity cDNA Reverse Transcription Kit (Thermo Fisher, 4368814). qPCR was performed using a Power SYBRTM Green PCR Master Mix and a CFX96 Touch Real-Time PCR Detection System from Bio-Rad (Thermo Fisher, 4367659). The comparative Ct technique (2-△△Ct) was used to test each sample in triplicate, and relative expression levels were calculated. These values were then adjusted to the level of RPLP0 mRNA. Additional file 6 contains a list of the qPCR primer sequences(17).

**Confocal microscopy**

An automated imaging system from Molecular Devices called ImageXpress Micro, which has environmental control, was used to acquire images (Molecular Devices). Nine images were captured per well using two channels to capture the matching signal from the target protein labeled with a fluorescent antibody [Alexa fluor 488: excitation (ex), 472/30 nm; emission (em), 520/35 nm] and the Hoechst 33342-stained nuclei (DAPI channel: ex, 377/50 nm; em, 447/60 nm). Using laser focusing with a z-offset, epifluorescence pictures were captured with a 40× objective (ELWD Plan Fluor, NA 0.6, WD 2.7). Target protein images were obtained at a predetermined z-offset from the DAPI channel after nuclear images. To reduce the amount of time, take to acquire an image of each sample, the imaging procedure was speed-optimized. The focus procedure made it possible to photograph one 96-well plate in around 1 h. A minimum of nine photos per well were required to provide enough cells, according to the optimization of the image acquisition procedure. Automated photography enables the representation of paired fields in the first and second images. In the primary analytic method used for screening, the Transfluor module of the MetaXpress program was employed to analyze the obtained images.

**GCase, Chitotriosidase activity, receptor activator of nuclear factor kappa-Β ligand (RANKL) and osteoprotegerin (OPG) enzyme-linked immunosorbent assays (ELISAs) analysis**

We used Glucosylceramidase activity assay kit (cat. ab273339, Abcam) to measure GCase activity and Chitotriosidase (CHIT) activity assay kit (cat. ab241009, Abcam) to measure CHIT activity, respectively, in monocytes collected from patients with GD, according to the manufacturer’s protocol. RANKL and OPG protein were quantified from the plasma by using Human ELISA Kit (ab213841 and ab189580, Abcam). The resultant Colorimetric and fluorescence (RANKL: OD 450nm; OPG OD 450nm; GCase activity: ex/em 360/445 nm; CHIT activity: ex/em, 320/445 nm) was measured using a SpectraMax iD3 microplate reader (Molecular Devices), and data processing was performed using SoftMax Pro 7.1 software (Molecular Devices).

**Assessing GBA1, calnexin, NLRP3, and ASC staining using flow cytometry**

We used a Foxp3/Transcription Factor Staining Buffer Set (eBioscience, Thermo Fisher) for cell fixation (2 × 10^6^ cells/ml) and permeabilization. Then, the cells were washed twice in phosphate-buffered saline (PBS) before blocking with 1% bovine serum albumin for 30 min at room temperature. Next, the cells were incubated for 30 min at 4°C following the addition of anti-GBA antibody (Abcam, #96256, [RRID: AB_10677756](https://antibodyregistry.org/search.php?q=AB_10677756),1:200), Calnexin antibody (CST, #2433, [RRID: AB_2243887](https://antibodyregistry.org/search.php?q=AB_2243887), 1:200), NLRP3 rabbit monoclonal antibody (CST, #13158, [RRID: AB_2798134](https://antibodyregistry.org/search.php?q=AB_2798134), 1:100), or ASC antibody (Santa Cruz Biotechnology, # sc-22514-R, [RRID: AB_2174874](https://antibodyregistry.org/search.php?q=AB_2174874),1:200). After three PBS washes, fluorescence-conjugated secondary antibodies (Alexa Fluor 488, Sigma–Aldrich, #16-237, [RRID: AB_436053](https://antibodyregistry.org/search.php?q=AB_436053), 1:200 or Alexa Fluor 647, Abcam, #150079, [RRID: AB_2722623](https://antibodyregistry.org/search.php?q=AB_2722623), 1:200) were added and incubated for 45 min at room temperature. Then, 400 µL of 1× binding buffer was gently mixed with the cell solution, incubated at 4^o^C for 5 min, and the cells were washed twice in PBS. Finally, we used the APC channel of a CytoFLEX flow cytometer (Beckman) to detect the stained cells. We used CytExpert 2.4 software (Beckman) to examine the cells and determine the percentages of cells positive for the various dyes.

**Ultra-performance Liquid chromatography-tandem mass spectrometry (UPLC-MS/MS) for Lyso-GB1**

UPLC-MS/MS was performed using a XEVO TQD triple-quadrupole mass spectrometer (Waters, Millford, MA) in positive ion mode. Aliquots (5 μL) of the samples were injected into an analytical column (ACQUITY UPLC CSH C18, 2.1 × 50 mm, 1.7 μm) with gradient elution using mobile phase A (30% acetnitrile, 70% water, 0.1% formic acid) and mobile phase B (50% acetonitrile, 50% isopropanol, 0.1% formic acid) at a flow rate of 0.8 mL/min at 55°C. The following gradient conditions were used: 1-70% B from 0-1.0 min; 70-75% B from 1.0-1.5 min; 1% B from 1.5-2.0 min. Data were acquired for 1.6 min during sample infusion. The total run time for one plate was approximately 3 hours.
