# Supplementary figures and images for: GBA1 as a risk gene for osteoporosis in the specific populations and its role in the development of Gaucher disease
Source: Orphanet J Rare Dis. 2024 Apr 4;19:144. doi: 10.1186/s13023-024-03132-x (PMC10993575; doi:10.1186/s13023-024-03132-x)

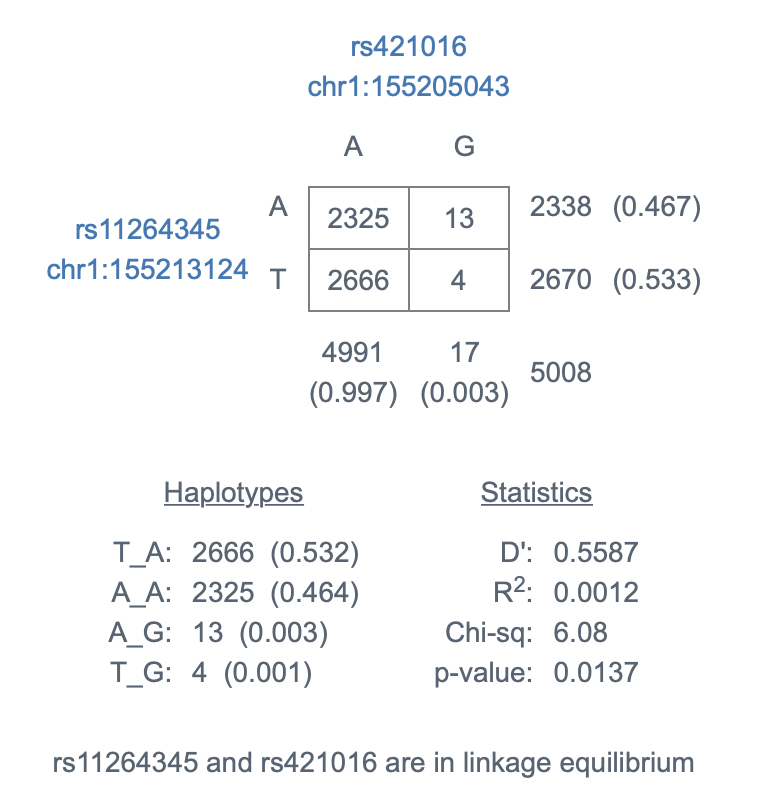

Supplement: Supplementary file 5 — Additional file 5: Linkage equilibrium between rs11264345 and rs421016. [file 13023_2024_3132_MOESM5_ESM.png]
