# Supplementary material for: GBA1 as a risk gene for osteoporosis in the specific populations and its role in the development of Gaucher disease
Source: Orphanet J Rare Dis. 2024 Apr 4;19:144. doi: 10.1186/s13023-024-03132-x (PMC10993575; doi:10.1186/s13023-024-03132-x)
Supplement: Supplementary file 6 — Additional file 6: The primer information for quantitative PCR. [file 13023_2024_3132_MOESM6_ESM.docx]

Additional file 6. The primer information for quantitative PCR

| Primer names | Primer sequences (5′–3′) |
| --- | --- |
| CTSK-F | CAGTGAAGAGGTGGTTCAGA |
| CTSK-R | AGAGTCTTGGGGCTCTACCTT |
| ACP5-F | CGGCCACGATCACAATCT |
| ACP5-R | GCTTTGAGGGGTCCATGA |
| NFATc1-F | AGAATTCGGCTTGCACAGG |
| NFATc1-R | CTCTGGTGGAGAAGCAGAGC |
| MMP9-F | GAACCAATCTCACCGACAGG |
| MMP9-R | GCCACCCGAGTGTAACCATA |
| OCSTAMP-F | CACGCTCACGGTCAAGTATG |
| OCSTAMP-R | TAGGAGCTGTGGACGGAGAG |
| ATF4-F | ATGACCGAAATGAGCTTCCTG |
| ATF4-R | GCTGGAGAACCCATGAGGT |
| CHOP-F | GGAAACAGAGTGGTCATTCCC |
| CHOP-R | CTGCTTGAGCCGTTCATTCTC |
| TRIB3-F | AAGCGGTTGGAGTTGGATGAC |
| TRIB3-R | CACGATCTGGAGCAGTAGGTG |
| XBP1-F | CCCTCCAGAACATCTCCCCAT |
| XBP1-R | ACATGACTGGGTCCAAGTTGT |
| GRB78-F | GAAAGAAGGTTACCCATGCAGT |
| GRB78-R | CAGGCCATAAGCAATAGCAGC |
| RPLP0-F | TCTACAACCCTGAAGTGCTTGAT |
| RPLP0-R | CAATCTGCAGACAGACACTGG |
| HPRT1-F | TGACCTTGATTTATTTTGCATACC |
| HPRT1-R | CGAGCAAGACGTTCAGTCCT |
